# The association between the Mediterranean Diet and the prime diet quality score and new-diagnosed irritable bowel syndrome: a matched case-control study

**DOI:** 10.3389/fmed.2025.1529374

**Published:** 2025-03-12

**Authors:** Ghazal Baghdadi, Marzieh Feyzpour, Seyedeh Atiye Shahrokhi, Roksaneh Amiri, Mehran Rahimlou

**Affiliations:** Department of Nutrition, School of Public Health, Zanjan University of Medical Sciences, Zanjan, Iran

**Keywords:** irritable bowel syndrome, Mediterranean Diet, Prime Diet Quality Score, case-control, nutrition

## Abstract

**Background:**

Irritable bowel syndrome (IBS) is a prevalent functional gastrointestinal disorder with multifactorial etiology. Dietary patterns, including the Mediterranean Diet (Med-Diet) and the Prime Diet Quality Score (PDQS), may play a role in IBS risk. This study examined the association between adherence to the Med-Diet and PDQS and new-diagnosed IBS in an Iranian population.

**Methods:**

A matched case-control study was conducted on 170 newly diagnosed IBS patients and 340 age- and sex-matched controls recruited from outpatient clinics in Zanjan, Iran. Dietary intake was assessed using a semiquantitative food frequency questionnaire. The Med-Diet score and PDQS were calculated, with higher scores indicating better diet quality. Conditional logistic regression was used to determine the odds of IBS across quartiles of Med-Diet and PDQS, adjusting for sociodemographic and clinical factors.

**Results:**

Higher adherence to the Med-Diet was associated with 51% lower odds of IBS (OR: 0.49; 95% CI: 0.30–0.73, *P* < 0.001) in the highest quartile compared to the lowest. Similarly, participants in the highest PDQS quartile showed a significantly 59% lower odds of IBS (OR: 0.41; 95% CI: 0.26–0.51, *P* < 0.001) compared to the lowest quartile. Both associations remained significant after adjusting for potential confounders, including total energy intake. These findings highlight the potential clinical relevance of dietary quality in IBS prevention.

**Conclusion:**

Higher adherence to the Med-Diet and a higher PDQS were both inversely associated with IBS risk. Specifically, individuals with higher Med-Diet scores and higher PDQS scores had a lower risk of developing IBS compared to those with lower adherence or scores. These findings suggest a potential role of these dietary patterns in modulating IBS risk, although causal relationships cannot be established from this study.

## 1 Introduction

Irritable bowel syndrome (IBS) is a common and chronic functional gastrointestinal disorder characterized by symptoms such as abdominal pain, bloating, and altered bowel habits, including diarrhea, constipation, or alternating patterns of both (1, 2). The global prevalence of IBS is estimated to be between 10 and 15%, with a higher incidence observed among women and young adults (1, 3, 4). IBS is classified under functional gastrointestinal disorders because structural abnormalities are often absent, and instead, symptoms are thought to arise from complex interactions between the central nervous system and gastrointestinal tract, frequently referred to as the gut-brain axis (5, 6). This intricate relationship is further influenced by factors such as low-grade inflammation, altered gut motility, hypersensitivity, and gut microbiota composition (7). Dietary habits have emerged as a significant factor in the modulation of IBS symptoms, with dietary triggers reported by up to 84% of IBS patients (8, 9). Various diets, including the low-FODMAP diet and gluten-free diet, have been proposed to mitigate symptoms in individuals with IBS; however, adherence to these restrictive diets is often challenging and may lead to nutritional deficiencies if not properly managed (9).

The Mediterranean Diet (Med-Diet), traditionally associated with countries bordering the Mediterranean Sea, is characterized by high consumption of fruits, vegetables, whole grains, legumes, fish, and healthy fats, particularly olive oil. While Iran is not geographically located in the Mediterranean basin, it shares several dietary characteristics with Mediterranean countries, such as high consumption of plant-based foods, legumes, and healthy oils (e.g., olive oil). Furthermore, recent studies have indicated that adherence to the Med-Diet is associated with a reduced risk of chronic diseases, including cardiovascular disease, diabetes, and gastrointestinal disorders, in diverse populations worldwide (10, 11). In Iran, despite not being part of the Mediterranean region, there is growing interest in the potential benefits of Mediterranean-style eating patterns. Previous studies have shown that diets similar to the Med-Diet, focusing on plant-based foods and healthy fats, may improve health outcomes in Iranian populations as well (12). For instance, Iranian studies have suggested that adherence to Med-Diet principles can improve metabolic health and reduce the risk of chronic conditions, including obesity and inflammatory diseases. Given the growing body of evidence supporting the Med-Diet’s benefits beyond Mediterranean countries, we chose to investigate its association with newly diagnosed IBS in an Iranian population, as it may offer a promising dietary approach for IBS prevention and management (13, 14).

Several studies have highlighted the potential of the Med-Diet to positively influence gastrointestinal health and associated symptoms. For instance, the Med-Diet’s emphasis on fiber-rich plant foods supports healthy gut microbiota diversity, a factor often compromised in IBS patients (15, 16). Moreover, the inclusion of omega-3 fatty acids from fish and monounsaturated fats from olive oil has been associated with reduced intestinal inflammation, which may play a role in modulating IBS symptoms (17). However, findings regarding the effectiveness of the Med-Diet in managing IBS remain inconsistent (10).

In addition to the Med-Diet, the Prime Diet Quality Score (PDQS) has been developed as a comprehensive tool to assess overall diet quality based on the consumption of nutrient-dense, minimally processed foods. Unlike the Med-Diet, which is regionally and culturally specific, PDQS offers a globally adaptable assessment of diet quality, encompassing a broad spectrum of food groups and nutrients (18). PDQS scoring is based on beneficial food groups, including fruits, vegetables, whole grains, nuts, and legumes, while limiting processed foods and sugars, thereby providing an accessible dietary framework applicable across diverse populations (19). Although research linking PDQS to IBS is limited, there is substantial evidence supporting the association between higher diet quality, as reflected by PDQS, and improved gastrointestinal health outcomes (20). Studies indicate that nutrient-dense diets may influence gut health through several mechanisms, including enhancing microbiota diversity and reducing inflammatory responses (21).

By using a matched case-control design, we intend to investigate whether higher adherence to the Mediterranean Diet and higher PDQS are associated with a lower risk of IBS. This study hypothesizes that individuals adhering more closely to the Med-Diet and achieving higher PDQS will demonstrate a reduced likelihood of IBS. These findings may provide valuable insights into dietary patterns associated with IBS risk, although causation cannot be inferred. Ultimately, findings from this study may contribute to evidence-based dietary guidelines, offering a holistic approach for IBS prevention and symptom management that is both sustainable and nutritionally adequate.

## 2 Materials and methods

### 2.1 Participants

This matched case-control study was conducted at several outpatient clinics in Iran from December 2023 to June 2024. The study included patients who were recently diagnosed with IBS by two gastroenterologists, adhering to the ROME IV diagnostic guidelines (22).

A control population was constituted by randomly selecting caregivers from patients who sought medical attention at the clinic but were free of any pathological conditions. This study was an individual matched case-control study and participants were matched in term of age and gender distribution. Case were 170 new diagnosed patients with IBS and control were 340 matched people to case group in a control to case ratio of 2:1. The protocol of the research was approved by the Ethics Committee of Tehran University of Medical Sciences (Ethics No: IR.TUMS.DDRI.REC.1402.026). All participants signed voluntary written consent letter. Also, all methods were performed in accordance with the Declaration of Helsinki guidelines and regulations.

### 2.2 Matching process

The matching process aimed to ensure that cases and controls were comparable with respect to age and gender, minimizing potential confounding from these variables. For each case, two controls were selected from the control population based on the following criteria:

1. **Age Matching:** Controls were selected to be within ±2 years of the case’s age.
2. **Gender Matching:** Each case was matched to two controls of the same gender.

### 2.3 Handling mismatches

During the matching process, some cases initially lacked exact matches for both age and gender due to limited availability in the control population. in such instances, broader matching criteria were applied:

• Age matches were extended to ±5 years if ±2 years were unavailable.
• If no exact gender matches were found, unmatched cases were included, and gender was adjusted for as a covariate in the regression analysis to account for potential mismatches.

These adjustments were documented, and sensitivity analyses were performed to ensure that mismatches did not substantially influence the study’s findings. This approach minimized bias while maximizing the use of available data.

### 2.4 Inclusion and exclusion criteria

We included adult patients aged 18–65 years, diagnosed with IBS, and following a non-restricted diet. Participants were excluded if they: (a) did not follow the study guidelines, (b) reported consuming more than 4500 or fewer than 800 calories per day, (c) exhibited severe fatigue, (d) were unable to provide answers to the study questions, (e) had a history of abdominal surgery, radiation, celiac disease, or other primary gastrointestinal disorders, (f) experienced gastrointestinal infections that masked IBS symptoms, (g) were pregnant or breastfeeding, or (h) adhered to specialized diets like vegetarianism or had significant weight changes in the previous year. Participants provided information about their: age, gender, marital status, weight, height, body mass index, occupation, education level, and smoking habits, including both active and passive smoking. This data was gathered through self-administered questionnaires.

### 2.5 Dietary intake evaluation

Dietary intake was assessed using a semiquantitative 168-item (23), which is validated for use in the Iranian population. To ensure consistency across participants, all FFQs were administered by trained interviewers using standardized protocols. Interviewers provided participants with detailed instructions, including examples of portion sizes illustrated with standardized reference images and food models, to minimize variability in reporting. The FFQ recorded the frequency and portion size of food and beverage consumption over the past year. However, it is important to acknowledge that FFQs are subject to recall bias, particularly in case-control designs, where participants may have difficulty accurately recalling their past dietary intake. Despite this limitation, FFQs remain a widely used method due to their ability to capture dietary patterns over a long period of time, and their use in this study was necessary to evaluate the association between dietary patterns and IBS.

Responses were converted to daily gram estimates for each food item using Nutritionist IV software, which was adapted for Iranian dietary habits. Rigorous interviewer training and uniform data entry procedures ensured consistency and reliability in dietary intake assessment across participants.

The Mediterranean Diet (Med-Diet) score was calculated using a method outlined by Trichopoulou et al. (24) A point was awarded for each of the following criteria: daily consumption of vegetables, whole grains, fish, legumes, and nuts at or above the sex-specific median intake; a MUFA-to-SFA ratio of grams greater than or equal to the sex-specific median; and daily consumption of meats and dairy products below the median intake. The overall Med-Diet score, ranging from 0 to 9, was determined by summing these component scores. A higher score indicates greater adherence to the Mediterranean Diet.

PDQS is a validated dietary quality index consisting of 14 healthy food groups and seven unhealthy food groups (25). The healthy food groups were inversely associated with disease risk, while the unhealthy food groups were positively associated with disease risk. The PDQS score was calculated based on the intake frequency of each food item within these groups. A point system was used to assign scores based on servings per week, with higher points for healthier food groups and lower points for unhealthy food groups. The overall PDQS score ranged from 0 to 42, with higher scores indicating a healthier diet.

### 2.6 Sample size

The sample size was determined through *a priori* power calculation to ensure sufficient power for detecting meaningful differences between cases and controls. The calculation was based on the Eslampour et al. (26) study examining dietary inflammatory index (DII) and IBS. Assuming an odds ratio of 0.5 for high adherence to the Mediterranean Diet or PDQS in reducing IBS risk, with a confidence level of 95% (α = 0.05) and 80% power (β = 0.20), the minimum required sample size was 150 cases and 300 controls, accounting for a 2:1 control-to-case ratio.

To account for potential dropouts or missing data, the sample size was increased to 170 cases and 340 controls. This ensured robust statistical power for detecting significant associations in the primary analysis and sufficient flexibility for subgroup analyses.

### 2.7 Irritable bowel syndrome symptom-related questionnaires

Participants with IBS completed validated questionnaires designed to assess gastrointestinal symptoms, such as the Bowel Symptom Questionnaire (27) and the irritable bowel syndrome Severity Scoring System (IBS-SSS) (28).

### 2.8 Psychological symptoms assessment

Participants completed the Visceral Sensitivity Index (VSI) (29), a validated questionnaire that assesses anxiety related to gastrointestinal symptoms, as well as the Hospital Anxiety and Depression Scale (HADS) (30), which measures current symptoms of anxiety and depression.

### 2.9 Other covariates

Chronic diseases were defined as any self-reported diagnosis of conditions such as diabetes, hypertension, cardiovascular disease, or other long-term health conditions. These were assessed through participant interviews and confirmed by medical records where available. About medication use, Participants were asked to report the use of any prescription or over-the-counter medications in the past 6 months. We categorized these medications based on therapeutic classes (e.g., antihypertensives, antidepressants, etc.).

Dietary supplement intake was assessed using a self-reported questionnaire asking participants about their use of vitamins, minerals, or other dietary supplements in the last 3 months. We categorized the supplements by type (e.g., multivitamins, fish oil, probiotics). Moreover, Regular meal patterns were defined based on the number of meals consumed daily, with “regular” being defined as having three or more meals per day. This was self-reported by participants in a structured questionnaire. Finally, physical activity was measured using the International Physical Activity Questionnaire (IPAQ), with participants reporting their activity levels in terms of moderate, vigorous, and walking exercises during the past week. The total physical activity score was calculated as the sum of these activities, classified into categories of sedentary, low, moderate, and high physical activity.

### 2.10 Statistical analysis

Data analysis was conducted using SPSS version 16. Continuous variables were summarized as mean and standard deviation, while categorical data were presented as frequency and percentage. The Kolmogorov-Smirnov test was employed to assess the normality of distribution for continuous variables. Independent t-tests were used to analyze demographic variables. Participants were divided into four groups based on their Med-Diet and PDQS scores. To compare general characteristics among these groups and between cases and controls, analysis of covariance (ANCOVA) was used for continuous variables, and the Chi-square test was used for categorical variables.

Confounders for the conditional logistic regression models were selected based on prior literature and clinical relevance. Factors known to influence both diet and IBS risk, such as age, sex, marital status, employment status, anxiety scores, education level, smoking habits, physical activity, and total energy intake, were included to minimize residual confounding. Additionally, dietary supplement use, medication use, chronic disease presence, and meal patterns were considered as covariates due to their potential impact on dietary quality and IBS outcomes. To address residual confounding, models were adjusted sequentially. The first model controlled for sociodemographic and behavioral factors, while the second model included additional dietary and clinical variables to account for potential confounding effects comprehensively.

To assess multicollinearity among predictors, we calculated the variance inflation factor (VIF) for all independent variables included in the logistic regression models, such as dietary patterns (Med-Diet and PDQS scores), energy intake, and other covariates. A VIF value greater than 5 was considered indicative of significant multicollinearity. In cases where multicollinearity was detected, we adjusted the model by either centering the variables, combining highly correlated predictors, or excluding redundant variables. This ensured the robustness of our findings and the reliability of the estimated coefficients. Additionally, we conducted sensitivity analyses to evaluate the robustness of our findings. Separate models were tested by varying the covariate adjustment sets, such as excluding total energy intake or excluding participants with extreme dietary intakes. These alternative models yielded consistent results, thereby supporting the robustness of the primary analyses. To explore potential interactions, we assessed whether psychological factors, such as anxiety and depression, modified the associations between dietary patterns and IBS. Interaction terms (e.g., dietary pattern × psychological factor) were included in the logistic regression models. Statistically significant interactions were identified using likelihood ratio tests, with a *p*-value threshold of <0.05. Significant interactions were further stratified to assess how the strength and direction of associations varied across levels of psychological factors. Statistical significance was defined as a *p*-value less than 0.05.

### 2.11 Sensitivity analysis

To assess the robustness of our findings and to evaluate the impact of residual confounding, we performed a sensitivity analysis to determine whether the observed associations between the Med-Diet score, the PDQS, and the health outcomes could be influenced by the inclusion or exclusion of specific confounders. The analysis involved re-evaluating the logistic regression models with variations in covariates. Specifically, we tested four different models: The primary model adjusted for age, sex, BMI, physical activity, and smoking status. The second model excluding depression as a covariate to assess its potential influence on the results. The third model including socioeconomic status to determine whether accounting for this factor affected the findings. Finally, a ‘leave-one-out’ approach where we removed one covariate at a time to check the stability of the results. The sensitivity analysis was conducted using logistic regression models in STATA version 15, and the ORs and 95% confidence intervals (CIs) were calculated for each model. We also conducted robustness checks using *E*-values to estimate the potential impact of unmeasured confounders on the observed associations.

The results of the sensitivity analysis were compared across the different models. A consistent association across all models would suggest the robustness of the primary findings. Any substantial changes in the odds ratios or *p*-values when excluding or including specific covariates would suggest that the observed relationships might be influenced by residual confounding and should be interpreted with caution.

## 3 Results

The baseline characteristics of participants in this study was summarized in Table 1. The mean age of the participants was 35.76 ± 9.65 years old and 42.35% of people were male. We found a significant differences between two groups in term of occupational status and physical activity level (*P* < 0.05).

**TABLE 1:** Distribution of 170 irritable bowel syndrome cases and 340 controls according to selected variables.

Variables	Mean ± SD or *N* (%)		*P*-value
	**Cases (*n* = 170)**	**Controls (*n* = 340)**	
Age (years)	35.76 ± 9.65	35.76 ± 9.66	1.00
BMI, kg/m^2^	24.76 ± 4.23	25.43 ± 3.89	0.532
Male (%)	72 (42.35)	144 (42.35)	1.00
**Education ^a^**			
Under diploma	27 (15.88)	57 (16.76)	
Diploma	78 (45.88)	137 (40.29)	0.281
Above diploma	65 (38.24)	146 (57.05)	
**Occupation ^a^**			
Employee	123 (72.35)	240 (70.58)	0.003
Unemployed	47 (27.65)	100 (29.42)	0.002
**Smoking ^a^**			
Yes	45 (26.47)	81 (23.82)	0.192
No	125 (73.53)	259 (76.18)	
**Anxiety level ^a^**			
Mild	104 (62)	275 (81)	
Moderate	28 (16.47)	42 (12.35)	<0.001
Severe	38 (21.53)	23 (6.65)	
**Depression level**			
Mild	89 (53)	262 (77)	<0.001
Moderate	52 (31)	60 (17.64)	
Severe	29 (16)	18 (5.36)	
**Physical activity ^a^**			
Low activity	93	178	
Moderate activity	58	104	<0.001
high activity levels	19	58	
**Bowel habit subtype, n (%)**			
IBS-C	42 (24.70)		
IBS-D	53 (31.17)		
IBS-M	28 (16.47)		
IBS-U	47 (27.66)		
Overall severity (0–20)	12.35 (5.22)		
Abdominal pain (0–20)	10.55 (4.83)		
Bloating (0–20)	13.67 (5.84)		
VSI score (0–75)	43.38 (19.46)		
IBS-SSS (0–500)	275.48 (73.16)		

^a^ = categorical variables. BMI, body mass index; IBS, irritable bowel syndrome; IBS-C, constipation-predominant IBS; IBS-D, diarrhea-predominant IBS; IBS-M, IBS with mixed bowel habits; IBS-U, IBS unclassified; IBS-SSS, Irritable Bowel Syndrome Severity Scoring System; VSI, Visceral Sensitivity Index.

The mean BMI of the participants in the case group was 24.76 ± 4.23 kg/m^2^ and in the control group was 25.43 ± 3.89, and a non-statistically significant difference was found between the two groups (*P* = 0.53). Our results indicated no statistically significant differences between cases and controls in terms of BMI (*P* = 0.53), smoking (*P* = 0.19), and education status (*P* = 0.28). These findings suggest that these factors may not have a strong or direct influence on IBS risk in our study population. The lack of association may reflect the relatively narrow range of these variables among our matched participants, as matching cases and controls by age and gender likely reduced variability in related sociodemographic factors.

The absence of significant differences in BMI, smoking, and education status between cases and controls provides important context for interpreting our findings. These results suggest that these variables may not play a substantial role in IBS risk within the studied population, although this does not rule out their potential relevance in other populations or under different conditions. The lack of significance for BMI aligns with previous studies that have found inconsistent or weak associations between body weight and IBS, possibly due to the multifactorial nature of the condition (31). Similarly, while smoking has been identified as a risk factor in some gastrointestinal disorders, its non-significant association in our study may reflect differences in exposure levels, cultural habits, or the relative importance of other factors such as dietary patterns. Education status also showed no significant difference, which could indicate that IBS is not strongly associated with socioeconomic factors in this context. However, it is important to note that the matched design of our study, particularly in age and gender, may have minimized the variability in these factors, potentially obscuring smaller effects.

However, in term of anxiety and depression levels, patients with IBS experienced higher levels of anxiety and depression (*P* < 0.001). Among the different subtypes of IBS, IBS-D was the most common among people in the case group (31.17%). The mean overall severity score and of IBS and abdominal pain score were 12.35 ± 5.22 and 10.55 ± 4.83 respectively.

Table 2 presents sociodemographic characteristics, dietary intake, and the prevalence of IBS subtypes across the quartiles of Med-diet and PDQS indices. Our results revealed that participants in the higher quartile of Med-diet and PDQS significantly consumed lower amounts of energy and macronutrients compared that subjects in the first quartile (*P* < 0.001).

**TABLE 2:** Biochemical measures, dietary factors, and disease severity among patients with different Med quartiles.

Variables	PDQS					Med-Diet				
**groups^1^**	**Q1 (*n* = 128)**	**Q2 (*n* = 128)**	**Q3 (*n* = 127)**	**Q4 (*n* = 127)**	**P^2^**	**Q1 (*n* = 128)**	**Q2 (*n* = 127)**	**Q3 (*n* = 127)**	**Q4 (*n* = 128)**	**P^2^**
Indexes (mean ± SD)	4.48 ± 2.34	14.74 ± 3.20	25.98 ± 2.96	34.66 ± 3.18	<0.001	0.96 ± 0.45	3.49 ± 0.77	6.08 ± 0.83	8.53 ± 0.88	<0.001
Energy (Kcal/day)	2415.80 ± 240.55	2354.46 ± 235.37	2289.37 ± 229.56	2301.19 ± 230.73	<0.001	2362.43 ± 237.63	2344.69 ± 228.54	2287.29 ± 220.27	2239.43 ± 223.18	<0.001
Carbohydrate (g/ day)	250.37 ± 22.40	247.81 ± 26.53	243.52 ± 19.64	247.51 ± 20.73	0.003	255.41 ± 17.48	245.28 ± 24.68	239.66 ± 18.43	246.12 ± 23.90	<0.001
Protein (g/d)	75.43 ± 6.19	78.47 ± 5.36	84.21 ± 10.37	87.13 ± 7.45	<0.001	76.23 ± 9.42	79.18 ± 11.48	82.65 ± 8.43	89.46 ± 13.46	<0.001
Total fat (g/ day)	81.39 ± 9.43	78.41 ± 8.27	83.45 ± 11.37	81.39 ± 8.37	<0.001	85.17 ± 10.17	82.75 ± 9.17	83.70 ± 9.46	80.25 ± 7.84	<0.001
MUFAs (g/d)	22.73 ± 4.36	23.66 ± 5.30	25.49 ± 4.33	28.71 ± 5.66	<0.001	22.30 ± 5.84	23.85 ± 4.95	25.44 ± 3.99	27.19 ± 4.83	<0.001
PUFAs (g/d)	11.45 ± 3.26	12.18 ± 2.85	13.55 ± 4.17	15.23 ± 4.33	<0.001	12.10 ± 3.15	13.46 ± 3.07	14.21 ± 2.91	16.33 ± 4.73	<0.001
SFAs (g/d)	41.37 ± 12.56	35.19 ± 14.39	31.49 ± 11.67	27.64 ± 17.54	<0.001	43.65 ± 14.77	37.52 ± 13.85	35.73 ± 12.19	26.19 ± 11.54	<0.001
High-fat dairy products (g/ day)	124.45 ± 6.33	97.82 ± 5.45	72.55 ± 7.45	49.32 ± 3.66	<0.001	119.30 ± 5.34	85.43 ± 6.15	77.19 ± 5.23	50.39 ± 4.26	<0.001
Low-fat dairy products (g/ day)	227.45 ± 5.36	237.27 ± 6.56	244.78 ± 7.29	273.94 ± 8.16	<0.001	219.69 ± 8.73	231.78 ± 9.23	255.46 ± 6.95	285.33 ± 7.11	<0.001
Fiber (g/ day)	17.25 ± 3.17	21.54 ± 2.17	23.64 ± 3.43	27.31 ± 4.19	<0.001	17.25 ± 1.86	19.53 ± 2.75	22.60 ± 3.11	24.88 ± 4.19	<0.001
Vegetables (g/ day)	228.35 ± 11.50	242.50 ± 10.42	301.43 ± 7.43	361.45 ± 9.23	<0.001	231.23 ± 9.47	266.54 ± 6.34	315.23 ± 8.56	354.90 ± 10.22	<0.001
Fruits (g/ day)	159.43 ± 4.12	173.39 ± 3.65	204.26 ± 4.15	253.66 ± 5.63	<0.001	148.31 ± 4.16	188.29 ± 6.35	239.43 ± 7.32	264.39 ± 5.32	<0.001
Legumes (g/ day)	37.25 ± 2.37	48.55 ± 1.77	63.54 ± 2.45	78.34 ± 3.15	<0.001	35.49 ± 2.66	41.34 ± 1.29	58.36 ± 1.55	72.44 ± 1.37	<0.001
Whole grains (g/ day)	85.43 ± 6.23	103.52 ± 7.46	115.49 ± 5.16	125.49 ± 6.32	<0.001	84.32 ± 4.68	97.35 ± 3.88	123.43 ± 4.67	136.49 ± 2.96	<0.001
Refined grains (g/ day)	265.33 ± 12.27	254.37 ± 9.43	228.37 ± 8.18	211.40 ± 10.24	<0.001	258.25 ± 11.35	243.83 ± 8.29	227.35 ± 9.12	205.19 ± 8.11	<0.001
Red and processed meats (g/ day)	44.20 ± 2.17	33.67 ± 2.76	28.40 ± 1.76	22.30 ± 2.19	<0.001	47.24 ± 2.37	36.47 ± 2.34	30.63 ± 1.85	24.77 ± 2.65	<0.001
Fishes and shrimps (g/ day)	9.68 ± 0.84	12.55 ± 1.07	16.32 ± 1.37	19.48 ± 1.17	<0.001	8.26 ± 0.74	12.65 ± 1.07	14.60 ± 0.74	21.37 ± 1.43	<0.001
Fast food (g/ day)	23.74 ± 1.26	18.22 ± 1.54	15.79 ± 1.70	11.44 ± 0.88	<0.001	25.12 ± 1.19	19.12 ± 1.23	14.76 ± 1.11	9.81 ± 0.77	<0.001
Total nuts and seeds (g/ day)	4.76 ± 0.64	6.19 ± 0.84	8.32 ± 0.55	12.19 ± 0.89	<0.001	5.13 ± 0.55	7.12 ± 0.74	9.23 ± 0.39	11.83 ± 0.41	<0.001
Current smokers (%)	33 (25.78)	30 (23.43)	29 (22.83)	34 (26.77)	0.37	35 (27.55)	31 (24.40)	30 (23.62)	30 (23.43)	0.432
**Anxiety level**										
Mild	80 (62.5)	87 (68)	98 (77)	114 (89.76)	<0.001	82 (64)	87 (68.52)	100 (78.74)	110 (85.93)	<0.001
Moderate	22 (17.2)	21 (16.40)	17 (13.4)	10 (7.9)		22 (17.2)	20 (15.74)	15 (11.81)	13 (10.15)	
Severe	26 (20.3)	20 (15.6)	12 (9.6)	3 (2.34)		24 (18.8)	20 (15.74)	12 (9.45)	5 (3.92)	
**Bowel habit subtype, n (%)**										
IBS-C	12 (9.37)	10 (7.81)	11 (8.66)	9 (7.08)	0.13	11 (8.59)	11 (8.66)	10 (7.87)	10 (7.81)	0.220
IBS-D	13 (10.15)	15 (11.71)	12 (9.44)	12 (9.44)		12 (9.37)	14 (11.02)	13 (10.23)	14 (10.93)	
IBS-M	6 (4.68)	8 (6.25)	7 (5.51)	7 (5.51)		7 (5.46)	7 (5.51)	7 (5.51)	7 (5.46)	
IBS-U	14 (10.93)	11 (8.59)	12 (9.44)	10 (7.81)		13 (10.15)	11 (8.66)	12 (9.44)	11 (8.59)	

^1^Values are means ± SD adjusted for age, sex, and energy except for Energy variable which was justified for age and sex.

^2^Derived from ANCOVA. PDQS, Prime diet quality score; Med-Diet, Mediterranean diet; P, *P*-value.

Also, we observed a significant higher intake of MUFA and PUFAs and lower amounts of SFAs among the subjects with good adherence from Med-diet and PDQS (*P* < 0.001). Moreover, higher adherence to both PDQS and Med-Diet scores was significantly associated with higher intake of low-fat dairy products, vegetables, fruits, legumes, fibers, whole grains, fishes and shrimps, nuts and seeds and lower intake of high-fat dairy products, refined grains, red and processed meats and fast foods (*P* < 0.001).

Table 3 presents ORs and 95% CIs of IBS across quartiles of Med-diet and PDQS indices. In crude model, increasing in Med-diet and PDQS as continuous variable was associated with lower odds of IBS [OR (95% CI): 0.51 (0.42–0.65), *P* < 0.001 for Med-diet and OR (95% CI): 0.39 (0.26–0.51), *P* < 0.001 for PDQS], indicating a strong association, though the direction of causality remains unclear. Furthermore, this correlation remained significant in the full adjusted models [OR (95% CI): 0.59 (0.46–0.75), *P* < 0.001 for Med-diet and OR (95% CI): 0.46 (0.30–0.59), *P* < 0.001 for PDQS]. On the other hand, when analyses were carried out with Med-diet and PDQS indices expressed as quartiles, we found that subjects in the higher quartile of Med-diet and PDQS compare than lower quartile experienced lower odds of IBS in both of the crude [OR (95% CI): 0.42 (0.25–0.64), *P* < 0.001 for Med-diet and OR (95% CI): 0.35 (0.24–0.48), *P* < 0.001 for PDQS] and full adjusted models [OR (95% CI): 0.49 (0.30–0.73), *P* < 0.001 for Med-diet and OR (95% CI): 0.41 (0.26–0.51), *P* < 0.001 for PDQS]. Our results revealed that higher adherence to the Med-Diet and PDQS was associated with substantial reductions in the odds of IBS. Specifically, adherence to the Med-Diet was linked to 51% lower odds of IBS (OR: 0.49; 95% CI: 0.30–0.73) in the highest quartile compared to the lowest. Similarly, adherence to the PDQS was associated with 59% lower odds of IBS (OR: 0.41; 95% CI: 0.26–0.51). These effect sizes are substantial and suggest that improvements in dietary quality may have significant implications for reducing IBS risk.

**TABLE 3:** Multivariate-adjusted ORs and 95% CIs for IBS in relation to Med-Diet and PDQS.

Med-Diet	Quartile1	Quartile2	Quartile3	Quartile4	P_*trend*_	Med-Diet (Continuous) OR (95% CI)	*P*-value
**Med-Diet (categorical) OR (95% CI)**							
Crude model	Ref	0.83 (0.63, 1.11)	0.69 (0.52,0.88)	0.42 (0.25, 0.64)	<0.001	0.51 (0.42, 0.65)	<0.001
Model 1	Ref	0.87 (0.66, 1.17)	0.73 (0.57, 0.95)	0.46 (0.26,0.70)	<0.001	0.55 (0.45,0.70)	<0.001
Model 2	Ref	0.93 (0.70,1.25)	0.75 (0.58, 0.99)	0.49 (0.30, 0.73)	<0.001	0.59 (0.46, 0.75)	<0.001
**PDQS**	**Quartile1**	**Quartile2**	**Quartile3**	**Quartile4**	**P_*trend*_**	**PDQS (Continuous)** **OR (95% CI)**	***P*-value**
**PDQS (categorical) OR (95% CI)**							
Crude model	Ref	0.75 (0.57,0.96)	0.58 (0.43,0.72)	0.35 (0.24,0.48)	<0.001	0.39 (0.26,0.51)	<0.001
Model 1	Ref	0.77 (0.58,1.04)	0.59 (0.43,0.74)	0.37 (0.26,0.48)	<0.001	0.43 (0.28,0.57)	<0.001
Model 2	Ref	0.81 (0.59,1.12)	0.62 (0.45,0.83)	0.41 (0.26,0.51)	<0.001	0.46 (0.30,0.59)	<0.001

Model 1: Adjusted for marital status, employment status, anxiety scores, education level, smoking, chronic disease, medication use, dietary supplement intake and regular meal pattern. Model 2: Further adjusted for physical activity, and total energy intake.

To explore potential heterogeneity in dietary associations, analyses were stratified by IBS subtypes, including IBS-D, IBS-C, mixed IBS IBS-M, and IBS-U. Higher adherence to the Mediterranean Diet was inversely associated with IBS-D (OR: 0.41; 95% CI: 0.25–0.59; *P* < 0.001) and IBS-M (OR: 0.50; 95% CI: 0.33–0.74; *P* = 0.002), but no significant association was observed for IBS-C or IBS-U. Similarly, a higher PDQS was associated with reduced odds of IBS-D (OR: 0.31; 95% CI: 0.19–0.49; *P* < 0.001) and IBS-M (OR: 0.47; 95% CI: 0.29–0.71; *P* = 0.001), while no significant associations were observed for other subtypes. These results indicate potential subtype-specific dietary effects.

### 3.1 Sensitivity analysis results

The results of the sensitivity analysis confirmed the robustness of our primary findings. When we calculated the OR using an alternative model specification that excluded depression as a covariate, the association between the Med-Diet score and the IBS remained statistically significant (OR = 0.52, 95% CI: 0.38, 0.81, *P* = 0.015). Similarly, when adjusting for socioeconomic status as an additional covariate, the association remained consistent (OR = 0.55, 95% CI: 0.39–0.85, *P* = 0.022), indicating that the relationship between Med-Diet score and the outcome is not highly sensitive to changes in model assumptions. Also, in term of PDQS, the significance of the results remained unchanged when we excluded depression (OR = 0.45, 95% CI: 0.28–0.66, *P* = 0.01) and socioeconomic status (OR = 0.46, 95% CI: 0.28–0.9, *P* = 0.015).

## 4 Discussion

In this matched case-control study among a sample of Iranian population, the results showed a significant inverse correlation between Med-diet score and PDQS with odds of IBS.

Some previous studies investigated the relationship between adherence from the Mediterranean dietary pattern and the risk of irritable bowel syndrome and reported conflicting results (10, 11). Prior investigations have posited a potential association between the Mediterranean dietary pattern and enhanced gastrointestinal functionality. Empirical evidence from two observational studies has demonstrated a discernible correlation between adherence to this dietary paradigm and a diminished incidence of functional gastrointestinal symptoms, thereby substantiating the hypothesis that the Mediterranean diet may be associated with improved gastrointestinal well-being (11, 32). Nonetheless, the paucity of rigorous randomized controlled trials precludes definitive conclusions regarding a causal relationship.

The inverse association between the Med-Diet score, the PDQS, and the risk of IBS observed in our study is noteworthy. This consistent finding across both dietary indices, despite their distinct cultural and dietary contexts, warrants further discussion. Although the Med-Diet is characterized by high consumption of fruits, vegetables, whole grains, and healthy fats, the PDQS was developed to assess overall diet quality based on a broader range of food groups. Despite the differences in cultural food practices and dietary patterns, both indices emphasize the importance of a plant-based, fiber-rich diet and the reduction of processed foods, which may explain the observed inverse association with IBS risk (33).

Previous studies have reported conflicting findings regarding the association between the Med-Diet and IBS. Chen et al. reported that a standard Med-Diet was not associated with IBS symptom severity. However, certain Med-Diet, such as high FODMAP-containing fruits and vegetables, were found to exacerbate IBS symptoms. Their findings suggest that a ‘one-size-fits-all’ approach with the Med-Diet may not be suitable for all IBS patients. Personalization of the Med-Diet, with a focus on identifying and avoiding foods that trigger IBS symptoms, may be necessary for patients with more severe symptoms. This aligns with our findings, where a high PDQS associated with adherence to a Med-Diet did not universally improve IBS symptoms in all participants (10). While the Mediterranean diet is widely considered beneficial due to its emphasis on fruits, vegetables, whole grains, and healthy fats, it is important to note that some components may not be suitable for all IBS patients. For example, high-FODMAP foods commonly found in the Mediterranean diet, such as certain fruits, vegetables, legumes, and dairy products, could exacerbate IBS symptoms in sensitive individuals. These components may need to be tailored or excluded to prevent symptom flare-ups. Therefore, achieving a high PDQS score, which reflects overall adherence to a Mediterranean dietary pattern, may not always lead to improved outcomes for IBS patients, especially if individuals are consuming foods that trigger their symptoms (34).

A study conducted by Zito and colleagues in Southern Italy, involving a cohort of 1134 individuals, uncovered a significant correlation between reduced adherence to the Mediterranean dietary pattern and an elevated incidence of irritable bowel syndrome and functional dyspepsia, particularly among younger demographics. The authors posited that a diminished consumption of Mediterranean diet staples may serve as a predisposing factor for the development of disorders characterized by the interplay between the gastrointestinal tract and the central nervous system within this specific population (11).

In a parallel study conducted by Agakidis and colleagues in Greece, a group of 1116 children were assessed. The researchers discovered a noteworthy correlation between consistent adherence to the Mediterranean dietary pattern and a reduced prevalence of gastrointestinal disorders influenced by the brain, including functional constipation, irritable bowel syndrome, and functional dyspepsia, as classified by the Rome III diagnostic criteria (32). One of the interesting points of these three studies was that one of these studies was conducted only on children from age 6–18 years old (32), in the other study it showed a significant relationship between the Mediterranean diet and IBS only among younger age group not adult (11), and in Chen ’s study., which was conducted on adults, no significant relationship was observed (10). However, our study conducted among the adult participants and we observed a significant inverse correlation between adherence from Med-diet and odds of IBS.

These discrepancies may stem from methodological differences, including dietary assessment tools, IBS diagnostic criteria, and population characteristics. For example, Zito et al. (11) employed a food frequency questionnaire tailored to a Mediterranean population, whereas Chen et al. (10) utilized a different scoring system for Med-Diet adherence. Additionally, age-related factors, such as gut microbiota composition and dietary behaviors, may contribute to variability in outcomes. Our study focused on an adult Iranian population and identified a significant inverse association, highlighting the need for region-specific and age-sensitive dietary recommendations.

The protective effects of the Med-Diet and PDQS on IBS may be explained through multiple mechanistic pathways. First, the Med-Diet, rich in fiber from fruits, vegetables, whole grains, and legumes, enhances gut microbiota diversity, which is often compromised in IBS patients (35). Increased dietary fiber promotes the growth of short-chain fatty acid (SCFA)-producing bacteria, such as *Faecalibacterium prausnitzii* and *Eubacterium* spp., which have been shown to improve gut barrier function and reduce intestinal permeability (36). SCFAs also exert anti-inflammatory effects by modulating immune responses and lowering pro-inflammatory cytokine levels, which are often elevated in IBS (37).

Polyphenols, abundant in Med-Diet staples such as olive oil and fruits, further contribute by reducing oxidative stress and inflammation through their antioxidant properties (10). These compounds have been shown to enhance the abundance of beneficial gut bacteria and decrease the population of pathogenic species, thereby improving gut health (38).

Although inflammation has been suggested as a contributing factor in IBS, it is important to distinguish IBS from IBD. IBS is primarily a functional gastrointestinal disorder characterized by symptoms such as abdominal pain and altered bowel movements, with minimal evidence of inflammation. Some studies have observed low-grade inflammatory markers in IBS patients, but these findings remain inconsistent (39, 40). In contrast, IBD, which includes Crohn’s disease and ulcerative colitis, involves chronic inflammation and visible damage to the intestinal lining. Therefore, while inflammation may play a role in the pathophysiology of IBS, the extent of this involvement is less pronounced compared to IBD and requires further investigation (41, 42).

The PDQS emphasizes nutrient-dense, minimally processed foods, which share similar gut-health benefits. Diets with high PDQS scores are associated with reduced markers of systemic inflammation, such as C-reactive protein (CRP), and increased microbial diversity (43). Moreover, both dietary patterns may improve the gut-brain axis function by modulating gut-derived neurotransmitters, such as serotonin, and reducing symptoms of anxiety and depression, which are strongly linked to IBS pathophysiology (44). These findings underscore the multifactorial nature of the Med-Diet and PDQS in influencing IBS risk and symptom severity through microbiota modulation, anti-inflammatory effects, and psychological health improvement. Further randomized controlled trials are needed to confirm these mechanisms and explore their clinical implications in IBS management.

In the recent randomized controlled trial, which conducted by Staudacher et al., researchers examined the effects of adherence from Med-diet for 6 weeks on clinical outcomes among the patients with IBS and they found a significant improvement in gastrointestinal symptom responders in the Mediterranean diet group than controls (45). The potential mechanisms underlying the observed associations between the Mediterranean diet and IBS are multifactorial. Importantly, the observed odds ratios reflect clinically meaningful reductions in IBS risk (54% for high adherence to the Mediterranean Diet and 59% for high adherence to PDQS). These effect sizes underscore the significance of dietary patterns in IBS prevention. In practical terms, promoting adherence to nutrient-dense dietary patterns such as the Mediterranean Diet and PDQS could serve as cost-effective strategies to mitigate IBS risk, particularly in populations where the burden of IBS is high.

Higher intake of fiber, antioxidants, and unsaturated fats in this diet likely enhances gut microbiota diversity and function, which has been implicated in the pathogenesis of IBS (46). The consumption of polyphenols from fruits and vegetables may also play a role in modulating inflammation and oxidative stress, both of which are thought to exacerbate IBS symptoms (47). Furthermore, the Mediterranean diet is known to promote a favorable balance of gut bacteria, including an increase in short-chain fatty acid (SCFA) producers, which have been associated with improved gut barrier function and reduced intestinal permeability (48). The Mediterranean diet can potentially alter the composition of the gut microbiome and the production of microbial metabolites (48). These changes may subsequently exert a direct impact on gut function. For instance, prior research has demonstrated that the Mediterranean diet can elevate the abundance of saccharolytic bacteria, recognized for their significance in butyrate production, such as Faecalibacterium prausnitzii, Eubacterium species, and Lachnospiraceae species (49, 50). Empirical evidence suggests that the Mediterranean diet can exert a microbiome-mediated anti-inflammatory influence in individuals who are genetically predisposed to inflammatory bowel diseases, such as Crohn’s disease. In addition, the Mediterranean diet may also modulate intestinal permeability, potentially through the intermediary role of the gut microbiota (51, 52).

Furthermore, the Mediterranean diet may ameliorate gastrointestinal symptoms by exerting a top-down regulatory influence on the gut-brain axis. In other words, by mitigating symptoms of anxiety and/or depression, the Mediterranean diet may alleviate the dysregulation of the gut-brain axis, thereby leading to a reduction in GI symptoms (53, 54). The anti-depressant features of Mediterranean diet have been shown in several clinical trials. The underlying mechanisms through which this dietary pattern influence anxiety and depression are likely multifaceted, involving immunological and oxidative stress mechanisms, the gut microbiome, neural plasticity, neurotransmitter synthesis, and mitochondrial function (55).

Our findings highlight the heterogeneity in dietary associations across IBS subtypes. Adherence to the Mediterranean Diet and PDQS showed significant inverse associations with IBS-D and IBS-M but not with IBS-C or IBS-U. These results align with previous studies suggesting that dietary factors may have subtype-specific effects based on underlying pathophysiological differences, such as altered motility in IBS-D versus IBS-C (56).

The stronger associations observed for IBS-D and IBS-M may reflect the anti-inflammatory and gut microbiota-modulating properties of the Mediterranean Diet, which could counteract mechanisms like increased intestinal permeability and dysbiosis often implicated in IBS-D (57). Further studies are needed to explore these mechanisms and confirm subtype-specific dietary recommendations.

While the Mediterranean Diet is widely recognized for its health benefits, including its potential role in reducing the risk of chronic diseases, it is important to note that the diet’s high-fiber content and the presence of certain high-FODMAP foods (such as legumes, onions, and certain fruits) can exacerbate symptoms in some individuals with IBS, particularly those with IBS-D (58). Current IBS management guidelines, such as those from the American College of Gastroenterology (ACG) (59) and the British Society of Gastroenterology (BSG) (60), recommend the low-FODMAP diet as a first-line approach, particularly for individuals with IBS-D, due to its ability to alleviate symptoms by reducing fermentable carbohydrates.

To reconcile these two approaches, future studies could explore how to modify the Mediterranean Diet for IBS patients. Potential modifications could include reducing or eliminating high-FODMAP foods (e.g., replacing high-FODMAP fruits and legumes with low-FODMAP alternatives), ensuring adequate fiber intake from easily digestible sources, and focusing on anti-inflammatory foods like olive oil and lean protein sources. These modifications would allow individuals with IBS to benefit from the overall health benefits of the Mediterranean Diet without exacerbating symptoms.

In terms of dietary quality, our study also demonstrates that higher PDQS scores were inversely associated with IBS risk, suggesting that a higher overall diet quality—characterized by nutrient-dense, minimally processed foods—may protect against IBS. According to our knowledge, no study has investigated the relationship between PDQS and risk of IBS. The PDQS, by emphasizing a diet rich in whole, unprocessed foods, overlaps significantly with the Mediterranean diet, which could explain the consistent findings across both dietary patterns (19). The decision to compare the Mediterranean diet with the PDQS in our study was motivated by the well-established reputation of the Mediterranean diet as a benchmark for healthy life style. However, despite its proven efficacy, the Mediterranean diet presents several challenges, particularly in low-income countries where its high cost and complex nature may hinder widespread adoption (61). Furthermore, its emphasis on specific nutrient ratios, such as saturated and monounsaturated fatty acids, poses practical difficulties for immediate clinical implementation. In contrast, the PDQS offers a more straightforward and accessible approach to assessing dietary quality. Its broader applicability and ease of use make it a valuable tool, especially in resource-limited settings where the adoption of complex dietary patterns like the Mediterranean diet may be impractical (62, 63). One of the mechanisms by which a diet with a high PDQS may reduce the risk of irritable bowel syndrome may be through a reduction in the severity of anxiety and depression. Haghighatdoost et al. in a cohort study among the healthy adults found that people who had a lower PDQS score had a 1.62 times higher risk for depression and a 1.5 times higher risk for anxiety than adults with higher PDQS score (64). Similar findings were reported in another study (65).

Several studies have indicated that psychological factors, particularly anxiety and depression, play a pivotal role in the pathophysiology of IBS (66). In our study, we observed a significant association between anxiety, depression, and IBS symptom severity, which aligns with previous research suggesting that mental health disorders can exacerbate gastrointestinal symptoms (67). Anxiety and depression may mediate the relationship between diet and IBS through various mechanisms, including altered gut motility, changes in gut microbiota, and dysregulation of the HPA axis. These findings suggest that managing psychological comorbidities in IBS patients could potentially improve both mental and physical health outcomes. Future studies should aim to further investigate the mediating effects of anxiety and depression on the diet-IBS relationship, employing advanced statistical models such as mediation analysis (68).

This study has several strengths that contribute to the understanding of dietary quality and its association with the risk of developing IBS. First, the use of a matched case-control design allowed for a robust comparison between newly diagnosed IBS patients and healthy controls, reducing confounding variables such as age, sex, and socioeconomic factors. Additionally, this study utilized validated dietary assessment tools, including the PDQS and Med-Diet adherence score, which offer comprehensive assessments of overall dietary quality and adherence, rather than relying solely on single nutrient or food analyses. This holistic approach provides valuable insights into the broader impact of dietary patterns on IBS risk and symptomatology. Another strength of our study is the use of sex-specific medians for calculating the Med-Diet score, which allows for a more nuanced reflection of dietary patterns. However, it is important to note that this approach may limit the comparability of our results with studies that have used the standard Med-Diet score calculation, which typically employs a combined median for both sexes. This difference in methodology may introduce variability in the interpretation and comparison of our findings with those from other populations or studies.

However, certain limitations must also be acknowledged. Firstly, this study employed a matched case-control design, which, while effective for identifying associations, does not allow for causal inferences. The retrospective nature of data collection introduces potential recall bias, particularly in self-reported dietary intake. Despite matching cases and controls on age and gender and adjusting for potential confounders, residual confounding cannot be entirely excluded. Future prospective cohort studies or randomized controlled trials are warranted to confirm the observed associations and establish causal relationships between adherence to dietary patterns, such as the Mediterranean Diet and PDQS, and IBS risk. These designs would allow for a clearer understanding of temporal relationships and the potential mechanisms underlying these associations.

Second, although the diagnosis of IBS in our study was based on self-reported symptoms, these symptoms were assessed using validated questionnaires, including the Rome IV criteria, which have been widely used for diagnosing IBS in clinical research. However, we acknowledge that the reliance on self-reported data may introduce some limitations in diagnostic accuracy. Unfortunately, clinical or laboratory diagnostic measures (such as endoscopic or biochemical tests) were not part of our study design, and symptoms were not independently verified through such measures. Given the multifactorial nature of IBS, this reliance on self-reported data should be considered when interpreting the results.

Third, this study utilized a semiquantitative FFQ to assess dietary intake over the past year. While the FFQ is a validated tool for capturing habitual dietary patterns, it is inherently prone to recall bias, particularly for long-term dietary assessments. Participants may have over- or underreported certain food intakes, potentially introducing measurement errors. This limitation may impact the accuracy of the observed associations, particularly for specific food groups or nutrients. To mitigate the effects of recall bias, the FFQ was administered by trained interviewers, and portion sizes were illustrated using standard reference images to improve accuracy. However, future studies should consider using alternative or complementary dietary assessment methods, such as 24-h dietary recalls, food diaries, or biomarkers of dietary intake (e.g., urinary or blood metabolites), to validate self-reported data and enhance the reliability of dietary assessments. These methods would allow for cross-validation and minimize reliance on memory for dietary reporting.

Another limitation is that some of the participants in the patient group may have been newly diagnosed with IBS These patients could have already made dietary adjustments in response to their gastrointestinal symptoms prior to their diagnosis. This phenomenon is particularly likely in this group, as individuals with undiagnosed gastrointestinal complaints may instinctively avoid certain foods or modify their diet to alleviate discomfort, which could confound the relationship between diet and IBS symptoms in this cohort. Therefore, it is possible that the observed dietary patterns may not accurately reflect the participants’ usual dietary intake before the onset of symptoms, introducing bias in our findings.

Moreover, the fifth limitation of this study is the lack of objective biomarkers or external validation measures to confirm adherence to the Mediterranean Diet or the PDQS. While the FFQ and scoring systems used are validated and widely accepted for dietary assessment, they rely on self-reported data, which can introduce bias and misclassification of dietary adherence. This limitation may affect the accuracy of the observed associations, particularly in distinguishing between high and low adherence groups.

Finally, in our manuscript, we posited that the observed inverse association between both the Med-Diet score and the PDQS and IBS risk may be mediated through mechanisms such as gut microbiota modulation and reduced inflammation, based on existing literature linking these factors to diet quality. However, we acknowledge that our study did not directly assess these biological mechanisms through microbiota analysis or inflammatory marker measurements. This is a limitation of our study, and future research should aim to include these direct measures to further validate the proposed mechanisms. While we provide a strong association between dietary patterns and IBS risk, the exact pathways underlying this relationship remain speculative and require further investigation.

Future studies should consider incorporating biomarkers of dietary adherence, such as plasma carotenoids, fatty acid profiles, or polyphenol metabolites, to objectively validate self-reported adherence to the Med-Diet score and the PDQS. These biomarkers could strengthen the reliability of findings by providing an external reference for dietary intake. Additionally, repeated measures of dietary intake over time would help address variations and improve data robustness. Lastly, the study sample was regionally specific, limiting the generalizability of these findings to other populations with varying dietary habits or cultural backgrounds.

## 5 Conclusion and future directions

In conclusion, this study demonstrates a significant inverse association between higher adherence to the Med-Diet score and the PDQS Scores with the likelihood of developing IBS, suggesting that diet quality may play a protective role in IBS risk. These findings underscore the potential association of dietary patterns rich in whole, unprocessed foods and healthy fats, as characterized by the Mediterranean diet, with reduced IBS risk. The observed 54–59% lower odds of IBS among the participants with higher adherence from Mediterranean Diet and PDQS highlight the clinical relevance of dietary quality in IBS prevention. Importantly, given the cultural and dietary variations across populations, our findings emphasize the need for culturally tailored dietary interventions that incorporate locally relevant food choices while promoting the core principles of a nutrient-dense, fiber-rich, and anti-inflammatory diet. Such tailored approaches could optimize dietary recommendations and improve the effectiveness of IBS prevention strategies across diverse populations.

While our study provides valuable insights into the associations between diet and IBS, it is important to note that the findings are cross-sectional in nature. This means we cannot definitively determine cause-and-effect relationships between dietary habits and IBS symptoms. To confirm causality and better understand the long-term effects of diet on IBS progression, future research should include longitudinal or intervention studies. These types of studies would allow researchers to track the impact of dietary changes over time, determine whether specific dietary modifications can reduce the risk of IBS symptom flare-ups, and explore how such changes might influence the severity or progression of the condition. Longitudinal studies could also help identify critical time points at which dietary interventions may have the greatest benefit, further contributing to the development of targeted dietary recommendations for IBS patients. Future studies are recommended to explore the causal relationship between dietary quality and IBS through longitudinal or intervention designs, assess the effects of diet on specific IBS subtypes, and investigate the role of additional factors such as gut microbiota modulation, inflammation, and psychological health in larger, diverse populations. Such research could further clarify the mechanisms by which diet influences IBS and refine dietary guidelines for IBS prevention and management.

## Data Availability

The raw data supporting the conclusions of this article will be made available by the authors, without undue reservation.
